# Effect of the PGD_2_-DP signaling pathway on primary cultured rat hippocampal neuron injury caused by aluminum overload

**DOI:** 10.1038/srep24646

**Published:** 2016-04-19

**Authors:** Jie Ma, Qunfang Yang, Yuling Wei, Yang Yang, Chaonan Ji, Xinyue Hu, Shaoshan Mai, Shengnan Kuang, Xiaoyan Tian, Ying Luo, Guojuan Liang, Junqing Yang

**Affiliations:** 1 Department of Pharmacology, Chongqing Medical University, the Key Laboratory of Biochemistry and Molecular Pharmacology, Chongqing 400016, China

## Abstract

In the present study, the agonists and antagonists of DP receptor were used to examine whether the PGD_2_-DP signaling pathway affects neuronal function. Primary cultured hippocampal neuron was prepared and treated with aluminum maltolate (100 μM) to establish the neuronal damage model. PGD_2_ and cAMP content was detected by ELISA. L-PGDS and DPs mRNA and protein expression were measured by RT-PCR and Western blotting, respectively. The aluminium-load neuron was treated with the DP_1_ agonist BW245C, the DP_1_ antagonist BWA868C, the DP_2_ agonist DK-PGD_2,_ and the DP_2_ antagonist CAY10471, respectively. Neuronal pathomorphology was observed using H-E staining. The cell viability and the lactate dehydrogenase leakage rates of neurons were measured with MTT and LDH kit, respectively. Ca^2+^ level was detected by Fluo-3/AM. In the model group, the MTT values obviously decreased; LDH leakage rates and PGD_2_ content increased significantly; L-PGDS, DP_1_ mRNA and protein expressions increased, and DP_2_ level decreased. BW245C reduced the Ca^2+^ fluorescence intensity and protected the neurons. DK-PGD_2_ increased the intensity of Ca^2+^ fluorescence, while CAY10471 had the opposite effect. In conclusion, contrary to the effect of DP_2_, the PGD_2_-DP_1_ signaling pathway protects against the primary cultured rat hippocampal neuronal injury caused by aluminum overload.

Neurodegenerative diseases (NDDs) of the central nervous system (CNS), including Alzheimer’s disease(AD), Amyotrophic lateral sclerosis (ALS), and Parkinson’s disease (PD), have increased dramatically in recent years, comprising 30% of the total cases of disease in humans. Though the current medical treatments have significantly improved the quality and length of life for NDD patients, NDDs remain a significant unresolved societal burden that afflicts millions of people worldwide.

Many studies have shown that the pathogenesis of the NDD_S_ includes ischemia, calcium overload, oxidative stress and inflammatory factors^1^,^2^,^3^,^4^,^5^,^6^. Among these factors, neuronal damage and apoptosis caused by inflammatory cytokines have been widely recognized.

Aluminum (Al), which is abundant in the crust, is omnipresent in everyday life and may enter the human body in many ways such as the environment, diet, or drugs. However, the physiological action of Al on humans is unclear. Since the first report of Al toxicity to humans at early 1970s, it has been identified that Al overload could cause severe brain damage and neurodegeneration^7^. In particular, Al was detected in senile plaques and neurofibrillary tangles in the brain tissues from AD patients^8^. Therefore, Al neurotoxicity could be involved in the degeneration of neurons and the production of Aβ peptide. As reported, the Al-induced neuronal injury is closely related to neuroinflammatory.

Inflammation is partially mediated by prostaglandins, which are mediated by the rate-limiting enzyme cyclooxygenase (COX). To date, studies on the significance of COX-2 and its metabolites in neural degenerative diseases suggest that Alzheimer’s disease is associated with the over-expression of COX-2^9^,^10^,^11^. Thus, COX-2 inhibitors have been widely used. Unfortunately, COX-2 inhibitors cause many side effects, such as renal toxicity^12^, decreased ulcer healing^13^ and adverse cardiovascular reactions^14^. To avoid such side effects, it is a key to determine the significance of the COX-2 downstream signaling pathway in nerve injury.

Prostaglandins (PGs) are a type of unsaturated fatty acid derivative produced from arachidonic acid catalyzed by COX^15^. Prostaglandin D_2_ is one of the most abundant PGs synthesized by PGDS in the brain^16^. PGDS includes L-PGDS (lipocalintype prostaglandin D synthase) and H-PGDS (hematopoietic prostaglandin D synthase). L-PGDS is highly expressed in the central nervous system^17^. Several studies have suggested that PGD_2_ may protect against neuronal lesions caused by multiple factors^18^, but it has also been reported that PGD_2_ can cause hippocampal neuron apoptosis^19^,^20^. PGD_2_ plays a role *via* activating on prostaglandin D_1_ and prostaglandin D_2_ receptors. Focusing on the PGD_2_-DP_s_ signaling pathway, this study aimed to evaluate the characteristics and significance of the changes of DP_1_ and DP_2_ in primary cultured hippocampal neuron treated with aluminum overload.

This experiment established the injury model of rat hippocampal neurons induced by aluminum overload *in vitro* and evaluated the characteristics of the PGDS-DP pathway by the methods of ELISA, PCR, Western blotting *etc*. Additionally, neurons were treated with the DP_1_ agonist BW245C, the DP_1_ antagonist BWA868C, the DP_2_ agonist DK-PGD_2_ and the DP_2_ antagonist CAY10471 to investigate the characteristics and significance of the changes of the PGDS-DP pathway. The results of our present study will provide a powerful experimental and theoretical basis for further research on the mechanism of the neuronal injury induced by aluminum overload, facilitating the development of drugs for the effective treatment of NND_S_.

## Results

### Primary cultured and identification of neurons

**Figure 1:**
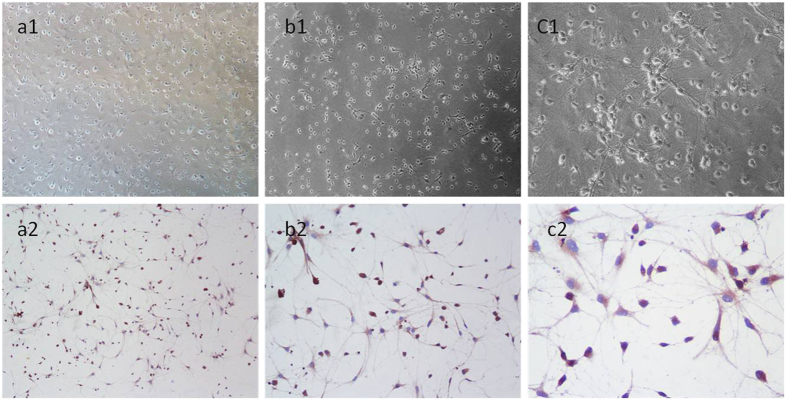
The morphology of primary cultured hippocampal neurons and NSE staining of neurons. (**a1,b1,c1**) Representative microscopic photographs show the morphology of hippocampal neurons after inoculation for 1d, 3d and 7d. Cultured 7 days later, adjacent neurons had developed to mutual cross-linked cell. Sections were pictured at 200× power. (**a2,b2,c2**) NSE staining showed that more than 95% of positive neurons existed. Sections were pictured at 100×, 200×, 400×, respectively.

Observed under inverted microscope, the neuronal soma was plumped with short processes after cultured 1 day. The neurons appeared oval or vertebral after 3 days, and they had become stereoscopic. For 7 days, the cultured neuronal bodies enlarged and the nervous process eruption and extension into reticulation. The purity of the hippocampus neurons was detected by neuron specific enolase (NSE) after cultured for 7 d. The bodies and axons of the positive cells were stained to be brown by NSE and nuclei were stained to be blue after counterstained with hematoxylin. One hundred cells were selected randomly to evaluate the number and the percentage of positive cells. Our results showed that there were more than 95% of positive cells (Fig. 1).

### Dose-dependent effect of Al (malt)_3_ and maltol on neuronal viability

**Figure 2:**
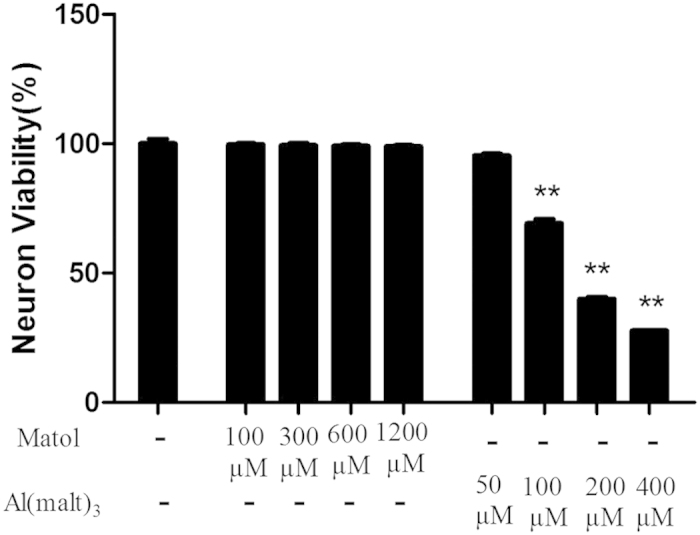
Dose-dependent effects of Al (malt)_3_ and maltol on neuronal viability detected by the method of MTT. The 100, 200 and 400 M of Al (malt)_3_ decreased the neuron viability significantly whereas the neuron viability at 50 μM decreased slightly. The concentration of Al^3+^ at 100 μM was suitable for the damage model. There was no considerable difference between the control group and the solvent control (maltol) (100–1200 μM) group. Values were mean ± SD of six individual experiments (n = 6, **P < 0.01 vs. control group, one-way ANOVA with Dunnett’s multiple comparisons).

The results showed that compared with the control group, the neuron viability decreased significantly in the Al^3+^-treated group at the concentration of 100, 200 and 400 μM with the neuron survival rates of 69.17%, 39.97% and 27.66%, respectively. However, the neuron survival rate was 95.47% at the concentration of 50 μM of Al (malt)_3_, which was no substantial difference compared with the control group. There was no considerable difference between the control group and the solvent control (maltol) (100–1200 μM) group. Considering each ion of Al is bound to three molecules of maltolate, 100 μM of Al (malt)_3_ and 300 μM of maltol were used in following experiments to evaluate the effects of PGD2-DP signaling on Al-load neuron (Fig. 2).

### Expression of DP_1_, DP_2_ and L-PGDS mRNAs and proteins in primary cultured rat hippocampal neurons

**Figure 3:**
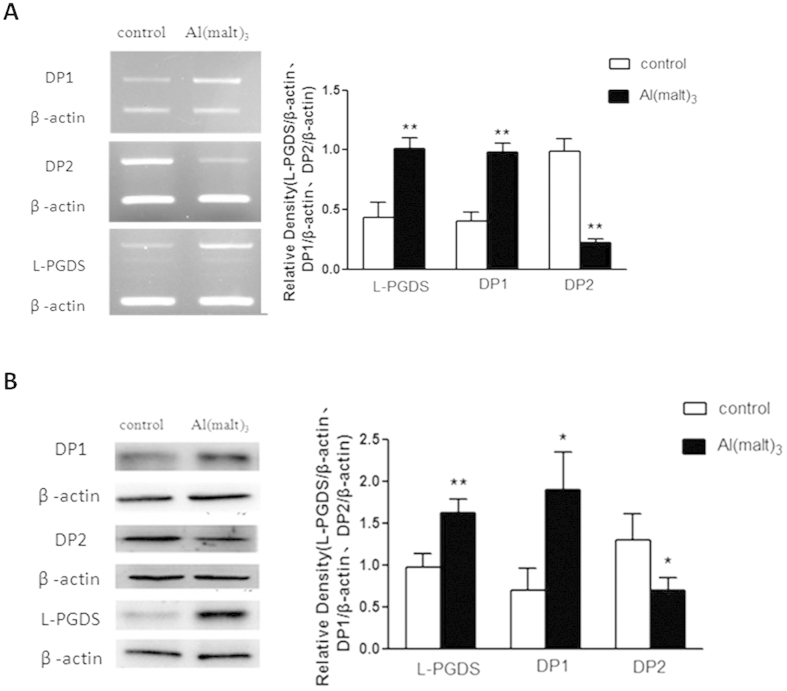
Levels of DP_1_, DP_2_ and L-PGDS in primary cultured rat hippocampal neurons detected by reverse transcription polymerase chain reaction. (**A**) The expressions of L-PGDS, DPs mRNA were measured by RT-PCR. The relative mRNA level of L-PGDS, DPs were standardized to endogenous β-actin mRNA for each sample. Al administration caused the significant increase of L-PGDS, DP_1_ levels and decrease of DP_2_ level compared with the control group. (**B**) The expressions of L-PGDS, DPs proteins were measured by WB. The relative protein levels of L-PGDS, DPs were standardized to endogenous β-actin protein for each sample. Al administration caused the significant increase of L-PGDS, DP_1_ levels and decrease of DP_2_ level compared with the control group. Values were mean ± SD (n = 3, **P < 0.01 vs. control group, one-way ANOVA with Dunnett’s multiple comparisons).

Compared with control group, the expression of L-PGDS and DP1 mRNA up-regulated 100% in the model group which was treated with Al (malt)_3_ (P < 0.01). By contrast, the expression of DP_2_ mRNA decreased significantly more than 70% in the model group (P < 0.01) (Fig. 3A).

The results of WB showed that compared with control group, the expression of L-PGDS proteins increased 50% (P < 0.01), and that of DP_1_ proteins nearly doubled in the Al^3+^-treated group (P < 0.05), however the expression of the DP_2_ proteins decreased 50% in the Al^3+^-treated group (P < 0.05) (Fig. 3B).

### The content of PGD_2_ was detected by Enzyme-linked Immunosorbent Assay

**Figure 4:**
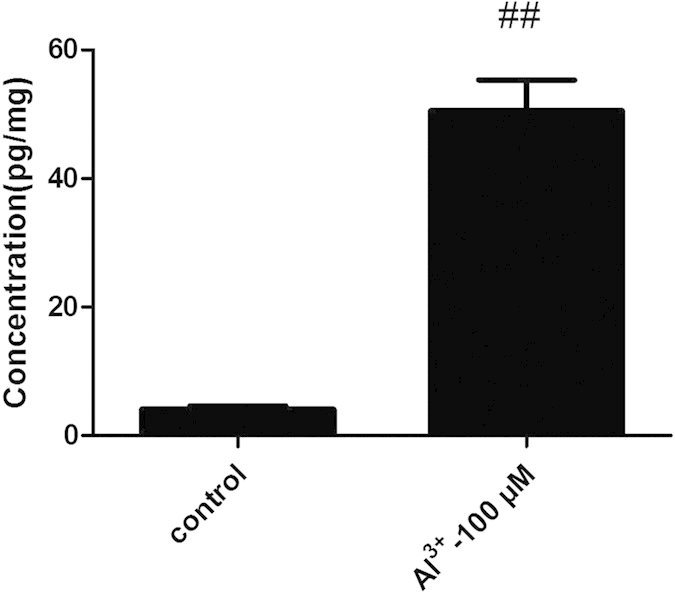
The content of PGD_2_ in each group detected by ELISA. The content of PGD_2_ raised significantly in the Al^3+^-treated group. Values were mean ± SD (n = 6, ^##^P < 0.01 vs. control group, Student’s t test).

In control group, the concentration of PGD_2_ was about 4pg/mg whereas it was approximately 50 pg/mg in Al^3+^-treated group which showed nearly 12 times of that in the control group. Compared with the control group, the content of PGD_2_ increased significantly in model group (P < 0.01) (Fig. 4).

### The change of neuron viability intervened by the DP agonists and antagonists

**Figure 5:**
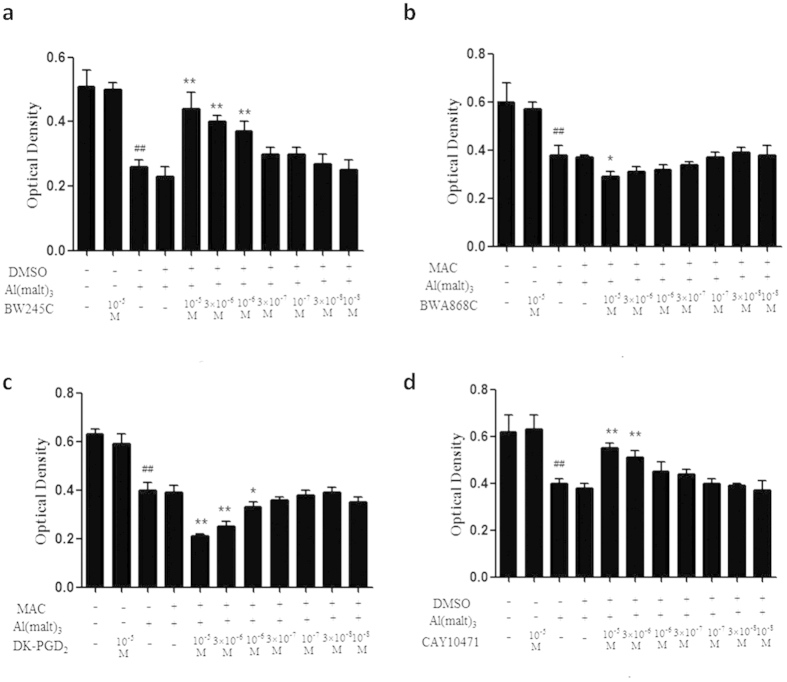
Effects of DP agonists and antagonists on the changes of neuronal viability caused by aluminium detected by the method of MTT. (**a**) BW245C and (**d**) CAY10471 increased neuron viability in a concentration dependent manner in Al^3+^-treated group, whereas (**b**) BWA868C and (**c**) DK-PGD_2_ decreased neuron viability in Al^3+^-treated group. Values were mean ± SD of eight individual experiments (n = 8, ^##^P < 0.01 compared with control group, *P < 0.05 and **P < 0.01 compared with Al^3+^-treated group, respectively, one-way ANOVA with Dunnett’s multiple comparisons).

About the concentrations of the agonists and antagonists of DP, for the observation of neuronal viability, seven concentrations (10^−5^, 3 × 10^−5^, 10^−6^, 3 × 10^−6^, 10^−7^, 3 × 10^−7^, 10^−8^, 3 × 10^−8^ M) of each agonists and antagonists of DP were used. The results showed that compared with the control group, the neuron viability decreased significantly in model group (P < 0.01) and that treatment of DP_1_ agonists (BW245C) at a concentration of 10^−6^, 3 × 10^−5^ and 10^−5^ M increased significantly the neuron viability compared with model group, whereas the viability of neuron treated with DP_1_ antagonist (BWA868C) of 10^−5^ M was decreased significantly (P < 0.01). Compared with model group, the neuron viability in the DP_2_ antagonist (CAY10471) (10^−6^, 3 × 10^−5^ and 10^−5^ M) -treated group was increased substantial. The administration of DP_2_ agonists (DK-PGD_2_) at a concentration of 10^−5^ and 3 × 10^−5^ M decreased neuron viability significantly (P < 0.01). There was no considerable difference between the control group and the solvent control group (Fig. 5).

We found each agonist and antagonist of DP had significant effects on neuron viability at a concentration of 10^−6^, 3 × 10^−5^ and 10^−5^ M, so we used the three concentrations of 10^−6^, 3 × 10^−5^ and 10^−5^ M of each agonist and antagonist of DP in lactate dehydrogenase leakage rate test.

### The change of LDH leakage rate intervened with the DP agonists and antagonists

**Figure 6:**
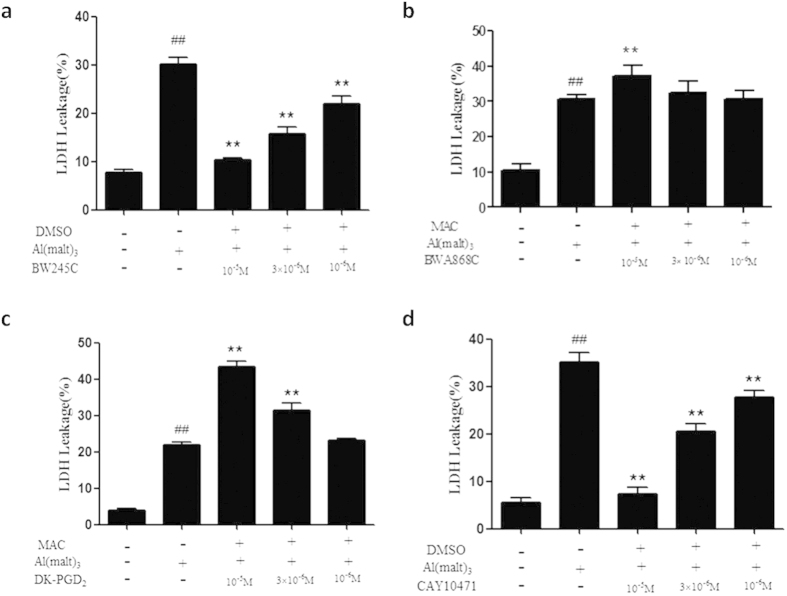
The change of LDH leakage rate intervened with the DP agonists and antagonists. (**a**) BW245C and (**d**) CAY10471 significantly decreased the LDH leakage rate in Al^3+^-treated groups. (**b**) BWA868C and (**c**) DK-PGD_2_ increased the LDH leakage rate in Al^3+^-treated groups. Values were mean ± SD of four individual experiments (n = 4. ^##^P < 0.01 compared with control group. **P < 0.01 compared with Al^3+^-treated group, one-way ANOVA with Dunnett’s multiple comparisons).

Compared with the control group, the solvent control group of primary cultured hippocampal neurons exhibited no considerable change in the LDH leakage rate, while the rate of LDH leakage rose significantly in the Al^3+^-treated group (P < 0.01). The DP1 agonist (BW245C) blunted the increase of leakage rate of LDH in Al (malt)_3_ –treated group in a concentration dependent manner (P < 0.01). The DP1 antagonist (BWA868C) of 10^−5^ M increased significantly the LDH leakage rate in Al (malt)_3_ –treated group (P < 0.01). The LDH leakage rate was increased significantly when treated with the DP_2_ agonists (DK-PGD_2_) at concentration of 10^−5^ M and 3 × 10^−5^ M (P < 0.01). The LDH leakage rate reduced significantly when treated with the DP_2_ antagonist (CAY10471) at different concentrations (10^−5^, 3 × 10^−5^ and 10^−6^ M) (P < 0.01) (Fig. 6).

Considering that there were a dose-dependent effect of each agonist and antagonist of DP at concentration of 10^−5^, 3 × 10^−5^ and 10^−6^ M on neuron viability and LDH leakage by Al, so, only the 10^−5^ M of the agonists and antagonists of DP were used in the observation of change of the neuronal pathology, cAMP content and Ca^2+^ fluorescence intensity in Al^3+^-treated neurons.

### The pathological change of primary cultured rat hippocampal neurons intervened with the DP agonists and antagonists

**Figure 7:**
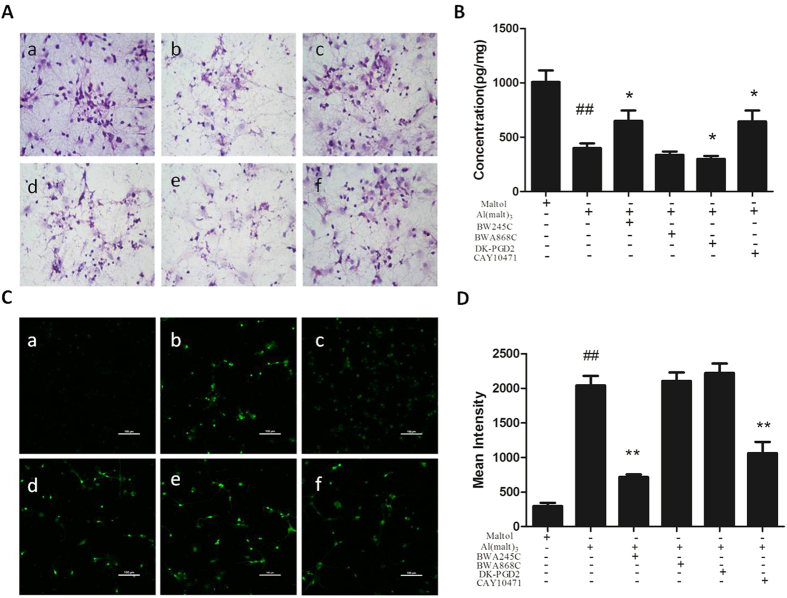
The effects of the DP agonists and antagonists on primary cultured rat hippocampal neurons. (**A**)The picture showed the number and structure of neurons in (a) control group and (b) Al^3+^-treated group. (c) BW245C- treated group and (f) CAY10471-treated group raised the number and structure of neurons in model group, whereas (d) BWA868C-treated group and (e) DK-PGD_2_- treated group decreased the number and structure of neurons in the Al^3+^-treated group. Sections were pictured at ×400. (**B**) The intervention effect of DP on the cAMP level in the Al^3+^-treated group. Values were mean ± SD of six individual experiments. (n = 6, ^##^P < 0.01 *vs*. control group. *P < 0.05 *vs*. Al-treated group, one-way ANOVA with Dunnett’s multiple comparisons) (**C**) The picture showed the Ca^2+^ fluorescence of neurons in (a) control group and (b) model group, (c) BW245C and (f) CAY10471 decreased the Ca^2+^ fluorescence of neurons in model group. (**d**) BWA868C and (e) DK-PGD_2_ increased the Ca^2+^ fluorescence compared with model group (×400). (**D**) Summary graph showed the intensity of Ca^2+^ fluorescence relative levels in DP agonists and antagonists groups against model group. Values were mean ± SD of ten individual experiments (n = 10. ^##^P < 0.01 compared with control group. **P < 0.01 compared with Al^3+^-treated group, one-way ANOVA with Dunnett’s multiple comparisons).

The structures of primary cultured hippocampal neurons were clear, complete and the protrusions of neurons were interwoven into the meshes in the control group. Compared with the control group, the number of neurons was substantial decreased and the protrusions were degenerated in the Al^3+^-treated group. Compared with the Al^3+^-treated group, the number of neurons increased and the structures recovered in BW245C-treated group. Treated with BWA868C, the neuronal injury was further aggravated compared with the Al^3+^-treated group. Treated with DK-PGD_2_, the hippocampal neurons almost exhibited karyopyknosis and disruption, whereas the bodies and nucleus of neurons were still clear and karyopyknosis compared with the Al^3+^-treated group. The neuronal injury in the Al^3+^-treated group was considerable reduced when treated with CAY10471 (Fig. 7A).

### The content of cAMP was detected by Enzyme-linked Immunosorbent Assay

The content of cAMP reduced significantly in the Al^3+^-treated group. Compared with the Al^3+^-treated group, the content of cAMP increased significantly when DP_1_ was activated or DP_2_ was inhibited (P < 0.05), whereas the content of cAMP showed a trend of decrease when DP_1_ was inhibited and the content of cAMP decreased significantly when DP_2_ was activated (P < 0.05) (Fig. 7B).

### Effect of DP interventions on the Ca^2+^ fluorescence intensity of primary cultured rat hippocampal neurons

The intensity of Ca^2+^ fluorescence was very weak in the control group. However, the intensity of Ca^2+^ fluorescence was significantly increased in the Al^3+^-treated group compared with the control group (P < 0.01) (Fig. 7C). Compared with the Al^3+^-treated group, the Ca^2+^ fluorescence intensity was reduced significantly by treatment of BW245C (P < 0.01). Treated with BWA868C or DK-PGD_2_, the intensity of Ca^2+^ fluorescence slightly increased in primary cultured hippocampal neurons. In contrast, compared with the Al^3+^-treated group, treatment of CAY10471 decreased significantly the intensity of Ca^2+^ fluorescence (P < 0.01) (Fig. 7D).

## Discussion

Aluminum accumulation may cause damage to cognitive function and central nervous system^21^. Maltol is a by-product of the hydrolysis of starch or sucrose, and it is also a common food additive^22^. Al (malt)_3_ can release Al^3+^ under physiological pH conditions *eg* in the gastrointestinal acid environment and facilitate the neurotoxicity^23^. Johnson *et al*. found that Al (malt)_3_ can cause apoptosis in primary cultured rat hippocampal neurons with time- and dose-dependent^22^. Our experiments showed that compared with the control group, MTT values were significantly decreased after treated with 100 μM Al (malt)_3_ for 24 hours in primary hippocampal neurons, while the LDH leakage rates were increased significantly. These results indicated that the injury model was successful.

COX and PGDS are the important rate-limiting enzymes for the synthesis of PGH_2_. PGDS has two subtypes, H-PGDS and L-PGDS. H-PGDS is mainly expressed in the placenta, lung, brain, and other tissues^19^,^24^. L-PGDS is the only trace protein in the lipocalin super family, which is mainly distributed in the brain and the reproductive organs and secreted into the cerebrospinal fluid, plasma and seminal fluid^25^,^26^. In recent years, it has been found that the expression of L-PGDS is related to cell apoptosis in the central nervous system^27^,^28^. The results of the present study showed that the expressions of L-PGDS mRNA and protein in the injury model induced by Al (malt)_3_ were significantly increased, suggested that L-PGDS may be involved in neuronal degeneration.

PGD_2_ is widely distributed and synthesized in the peripheral and central nervous systems. In the periphery, the main physiological functions of PGD_2_ contain the regulation of vascular diastolic and systolic pressure, and the inhibition of platelet aggregation^29^,^30^,^31^. In the brain, the main physiological functions of PGD_2_ contain the regulation of sleep, body temperature, olfactory function, sexual hormones and anti-anxiety effects^32^. In addition, PGD_2_ can significantly increase the contents of NGF (neuron growth factor) and BDNF (brain-derived neurotrophic factor), indicating that PGD_2_ may play a neuroprotective role in the CNS^30^. Our experimental results in this study showed that the PGD_2_ level increased significantly in the primary cultured rat hippocampal neurons, the main cause may be the intracellular calcium overload which sets out free radicals to activate phospholipase A2 (PLA2) which can produce more AA when Al is accumulated. At the same time, the expression of L-PGDS increased, and then the synthesis of PGD_2_ increased.

PGD_2_ acts as a potent positive modulator of DP receptors. There are two receptor subtypes for PGD_2_, DP_1_ receptor and DP_2_ receptor. DP_1_ is characterized by low expression, and it is mainly expressed in the hippocampus, cortex, hypothalamus and striatum of the brain tissue in addition to the peripheral circulation. Studies indicated that compared with the C57 mice, the susceptibility of C57 DP1 (−/−) mice to ischemia reperfusion injury was enhanced and the infarction area was significantly amplified^33^, suggesting that DP_1_ plays an obvious protective role in the ischemia reperfusion injury of brain. However, the protective mechanisms still unknown. DP_2_ is characterized by low expression, either, and it is widely distributed in the thalamus, cortex, hippocampus and other parts of the CNS. Studies reported that DP_2_ showed high expression and an excitotoxicity in normal hippocampal pyramidal neurons, and that DP_2_ agonist could significantly enhance the damage of the hippocampal CA1 and CA3 neurons caused by glutamate toxicity. In general, DP_1_ plays a protective role in the excitotoxicity and ischemic brain injury model, while DP_2_ accelerates the process of brain injury^19^.

In our study, we found that the content of PGD_2_ significantly increased after treated with aluminum in primary cultured rat hippocampal neurons as well as the expression of L-PGDS and DP_1_, whereas the expression of DP_2_ decreased. The results suggested that the PGD_2_-DP pathway may be involved in the injury of neurons. When DP_1_ was stimulated, the content of cAMP in the neurons and the release of Ca^2+^ increased, the barrier function of endothelial neurons enhanced^34^,^35^. However, research indicated that PGD_2_ promoted the aggregation of astrocytes in epileptic mice by activating DP_1_^36^. Liang *et al*.^19^ reported that BW245C can significantly reduce the mortality of neurons caused by the treatment of primary hippocampal neurons and hippocampal slices with NMDA results in excitation injury. Ahmad *et al*.^34^ found that BW245C significantly enhanced the brain damage and magnified the cerebral infarction area caused by NMDA. Saleem *et al*.^37^ found that the viability of primary cultured mouse cortical neurons damage induced by glutamate was improved after treated with BW245C, whereas the viability of neurons showed non-significant changed after treated with BWA868C. However, these results indicated that the specific effects of DP_1_ and DP_2_ on neuronal damage are still unclear. Therefore, in our experiment, we intervened in the PGD_2_-DP pathway with DP agonists and antagonists to clarify the importance of this pathway in the injury progress of primary cultured rat hippocampal neurons induced by aluminum. In our study, compared with the Al (malt)_3_ treated groups, the number of hippocampal neurons significantly increased after treated with DP_1_ agonist (BW245C). At the same time, protrusions were interwoven into the mesh and the MTT values increased greatly, In addition, the LDH leakage rate and the intensity of Ca^2+^ fluorescence decreased significantly. In the DP_1_ antagonist (BWA868C)-treated group, hippocampal neuronal bodies ruptured and neuronal structures were incomplete, meanwhile MTT values reduced and the LDH leakage rate increased significantly although the changes of the intensity of Ca^2+^ fluorescence were not obvious in the BWA868C-treated group. Our experimental results are consistent with that of the study of Saleem *et al*.^37^. Moreover, it proclaimed that the survival rates of neurons can be increased by BW245C, while the opposite results were observed by BWA868C, suggesting that the expression and activation of DP_1_ could reduce the injury susceptibility of hippocampal neurons to aluminum toxicity.

Studies also reported that DP_2_ receptors were activated coupled to Gi protein and inhibited cAMP levels, increasing Ca^2+^ in neurons^38^. In our experiments, we found that all of the neurons in the DK-PGD_2_ (DP_2_ agonist)-treated group underwent death compared with model group. These data also showed that MTT value decreased, LDH leakage rate increased significantly and the intensity of Ca^2+^ fluorescence rose up. Karyopyknosis and disruption of neurons significantly reduced in the CAY10471 (DP_2_ antagonist) -treated group, while MTT values increased, and LDH leakage rates reduced. These results in the present study suggested that DP_2_ may mediate the neurotoxicity of PGD_2_.

However, it has also been reported that the DP_2_ antagonist BAY-u3405 does not effect on the injury model induced by PGD_2_, and it is speculated that DP_2_ may not mediate the neurotoxicity of PGD_2_^39^. The contradictory effects of DP_2_ on neurons may be related to the neuron type and the degree of damage, and it needs further research in order to clarify its effect on neurons apoptosis in the over-expression DP_2_ or knockout mice. However, in this experiment, DK-PGD_2_ decreased the survival rates of neurons by activating DP_2_, whereas CAY10471 increased the survival rates by antagonizing DP_2_, indicating that the susceptibility of hippocampal neurons to aluminum neurotoxicity is increased by activating and expressing of DP_2_. This mechanism may be involved that its effect on DP receptor by regulating the Ca^2+^ signaling pathway, but the specific mechanism of the neural system is still unclear.

In conclusion, the expression of DP_1_ increased while DP_2_ decreased in the model induced by aluminum. DP_1_ expression and activation could decrease the injury susceptibility of hippocampal neurons to aluminum toxicity. The susceptibility of hippocampal neurons to aluminum neurotoxicity increased by activating and expressing of DP_2_. These results suggested that DP_1_ may protect the primary cultured hippocampal neuron from aluminum load damage, whereas DP_2_ may be harmful. DP can be considered as a potential candidate target for treatment of brain injury and neurodegenerative disease. However, considering the complex COX-2 downstream pathway, the complex regulation mechanism of DP in the central nervous system is worth our further study.

## Methods

### Animals

Rats were housed in the barrier housing facility, and it has in keeping with national standard “Laboratory Animal-Requirements of Environment and Housing Facilities”. The care of laboratory animal and the animal experimental operation have conforming to “Chongqing Administration Rule of Laboratory Animal”. The experimental procedures were approved by the animal laboratory administrative center and the institutional ethics committee of Chongqing Medical University (License number: SYXK YU 2012-0001) and also in accordance with the National Institutes of Health guidelines.

### Chemicals

AlCl_3_·6H_2_O (*Sinopharm Chemical Reagent Co., Ltd.,China*) and maltol (*Aladdin, USA*) were of analytical grade. Aluminum maltol solution (20 mM) was prepared by adding 1.513 g maltol and 0.483 g of AlCl_3_ ·6H_2_O into 200 ml of autoclaved PBS. The 20 mM maltol aluminum was stored at 4 °C until used with the methods^22^,^40^. BW245C and CAY1047 (*Cayman,USA*) were dissolved in the DMSO (*Sigma, USA*) to be 10 mM reserve liquid. BWA868C and DK-PGD_2_ (*Cayman,USA*) were dissolved in methyl acetate to be 3.5 mM reserve liquid^22^.

### Rat primary hippocampal neuron culture

Primary hippocampal neurons were prepared from E18 rat embryos and were immediately soaked with 75% ethanol. The hippocampus was isolated from the brain of each rat and the tissues were minced and digested with 0.125% trypsin at 37 °C for 20 min; digestion was stopped with the addition of 10% fetal bovine serum (FBS) (*Gibco, USA*). The neurons were centrifuged and suspended to a density of 1 × 10^6^/L in DMEM (*HyClone, USA*) with 10% FBS in it. The different volumes of neuronal suspensions were inoculated in culture flasks and coated with L-poly lysine (*Sigma, USA*) and cultured in a humidified 5% CO_2_ atmosphere at 37 °C. When the neurons adhered, the medium was changed to neurobasal medium (*Gibco, USA*).

### Neuron-specific enolase identification

Hippocampal neurons grown on glass cover slips were rinsed three times with PBS and fixed with 4% paraformaldehyde for 30 min at 4 °C and for 10 min at room temperature. The activity of endogenous peroxidase in the neurons was quenched with 3% H_2_O_2_ for 15 min. Goat serum (10%) was used as a blocking solution for 20 min at room temperature after three times washing. Then, neurons were incubated overnight at 4 °C with the appropriate dilutions of antibodies (NSE 1:50) (*Boston, China*). Afterward, the neurons were incubated with the second antibody (biotin-labeled goat anti-rabbit) for 30 min at 37 °C and with horseradish peroxidase-labeled avidin at 37 °C for 30 min. DAB (*ZSGB, China*) was used to analyze color development, and the samples were counterstained with hematoxylin, dehydrated with a series of graded alcohols, treated with xylene and sealed with neutral gum.

### Establishment of models

On the seventh day, hippocampal neurons were divided into the control group, four solvent groups and four Al^3+^-treated groups. PBS was added into the control group, 100, 300, 600, 1200 μM of maltol was added into the solvent group, respectively. While 50, 100, 200 and 400 μM Al (malt)_3_ were added into the model group, respectively. After the experiment, the most suitable concentration of Al (malt)_3_ was selected in following experiments^41^.

### PGD_2_ was detected by Enzyme-Linked Immunosorbent Assay (ELISA)

The rat hippocampal neurons were cultured in the flasks and divided into the control group and the Al^3+^-treated group until D7. After 24 h, neurons were collected after trypsin digestion. The levels of PGD_2_ was detected by ELISA kits (*Cloud-Clone Corp, USA*), following the manufacturer’s protocols.

### Reverse Transcription Polymerase Chain Reaction (RT-PCR)

To determine the expressions of L-PGDS, DP_1_, and DP_2_ mRNA in the control and the model groups, total RNA was extracted from the neurons using RNAiso Plus reagent (*Takara, China*) to generate cDNA templates by reverse transcription (RT) kit (*Takara, China*) following the manufacturer’s instructions, and then amplified by using the MIX PCR kit (*Cwbio, China*). PCR products were separated by 2% agarose gel electrophoresis and visualized by ethidium bromide staining. All the samples were normalized by the expression level of β-actin. The absorbance values of L-PGDS, DP1, DP2 and β-actin mRNA were measured with a Bio-Rad gel imaging analysis system (*Bio-Rad, USA*). The primer sequences for L-PGDS were: forward 5′-ATGTGCCAGACAGTGGTAGC-3′ and reverse 5′-TGGTCCTTGCTAAAGGTGATG-3′ (410 bp), DP1 (5′-TGAATGAGTCCTATCGCTGTC-3′ and 5′-GGTGATGTGCCTTTGGTAGAA-3′, 320 bp), DP2 (5′-CTTCCAAACCACAGCAACTC-3′ and 5′-CAGAGCATCAGGCAGACTC-3′, 326 bp), β-actin (5′-ACGGTCAGGTCATCACTATCG-3′ and 5′-GGCATAGAGGTCTTTACGGATG-3′, 155 bp).

### Western blotting

Neurons from each group were homogenized within 10 volumes of ice-cold homogenization buffer and centrifuged at 12,000 × g for 10 min at 4 °C. The supernatant was collected, and the protein concentrations were determined with a BCA protein assay kit (*Beyotime, China*). Twenty micrograms of protein was separated by sodium dodecyl sulfate polyacrylamide gel electrophoresis (SDS-PAGE) and then transferred to PVDF membranes (*Millipore, USA*). The membranes were blocked with 5% non-fat dry milk for 1 h at room temperature and then probed with specific primary antibodies, including anti-L-PGDS, DP_1_, DP_2_ (1:200; *Cayman, USA*), and β-actin (1:1000; *Boston, China*) overnight at 4 °C. The membranes were washed for three times in TBST and incubated with HRP-conjugated secondary antibodies at room temperature for 1 h, and then washed four times in TBST, protein signals were visualized by ECL (*Bio-Rad, USA*). Quantification of data and subsequent statistical analyses were performed with Image Lab.

### Detection of neuron viability

The primary cultured hippocampal neuronal viability was determined by 3-(4,5-Dimethyl-thiazol-2-yl)-2, 5-diphenyl-tetrazolium bromide (MTT) (*Sigma, USA*) assay. The neurons were plated in 96-well culture plates at a density of 1 × 10^5^ neurons/ml. The neurons were divided into the control group, the solvent control group, the Al^3+^-treated group and the intervention group. Different drugs including DP_1_ agonist (BW245C), DP_1_ antagonist (BWA868C), DP_2_ agonist (DK-PGD_2_), and DP_2_ antagonist (CAY10471) were added up into the intervention group respectively with various concentrations (10^−5^, 3 × 10^−5^, 10^−6^, 3 × 10^−6^, 10^−7^, 3 × 10^−7^, 10^−8^, 3 × 10^−8^ M) for 24 hours at 37 °C and 5% CO_2_. Then, the supernatant was discarded and 100 μl of 5 mg/ml MTT was added to each well. The media was carefully removed and the color was developed after incubation with 150 μl DMSO for 4 hours. Finally, absorbance (OD) was read at 570 nm by a micro plate reader (*BioTek, USA*)^42^.

### Lactate dehydrogenase (LDH) leakage rate measure

The cultured rat hippocampal neurons in the 24-well culture plate were cultured until D7 for drug treatment. The neurons were divided into the control group, the solvent control group, the Al^3+^-treated group and the intervention group. Different drugs including DP_1_ agonist (BW245C), DP_1_ antagonist (BWA868C), DP_2_ agonist (DK-PGD_2_), and DP_2_ antagonist (CAY10471) were added up into the intervention group respectively with various concentrations (10^−5^, 3 × 10^−5^, 10^−6^ M) for 24 hours at 37 °C and 5% CO_2_. Then, the LDH test kit was used according to the manufacturer’s instructions (*Beyotime, China*)^42^.

### Observation of pathological morphology

Cover slips (10 mm × 10 mm) were applied to the 24 wells of rat hippocampal neurons from cultured until D7 for drug treatment. The neurons were divided into the control group, the solvent control group, the Al^3+^-treated group and the intervention group. 10^−5^ M of BW245C, BWA868C, DK-PGD_2_, and CAY10471 were added up into the intervention group, respectively. HE staining was performed on cultured cells after 24 h as previously described in detail. In brief, cells were rinsed with PBS, fixed with 4% paraformaldehyde (PFA) for 30 min and then washed by PBS. The neurons were stained with Hematoxylin-Eosin; afterwards, they were dehydrated in alcohols. Morphological changes of the neurons were observed under an optical microscope (*Olympus, Japan*) after mounted by neutral resins.

### Content of cAMP detection by ELISA

The hippocampal neurons were divided into groups: the control group, the normal solvent group (300 μM of maltol), the normal intervention group (10^−5^ M BW245C, BWA868C, DK-PGD_2_, CAY10471) (*Cayman, USA*), the model group (100 μM Al(malt)_3_), the model solvent group (Al(malt)_3_ +10^−3^ M DMSO), and the model intervention group (Al(malt)_3_ + 10^−5^ M BW245C, BWA868C, DK-PGD_2_, CAY10471).

### Concentration of Ca^2+^ detection

The cultured rat hippocampal neurons in the special culture dish were cultured until D7 for drug treatment. The neurons were divided into the control group, the solvent control group, the Al^3+^-treated group and intervention group, Different drugs including DP_1_ agonist (BW245C), DP_1_ antagonist (BWA868C), DP_2_ agonist (DK-PGD_2_), and DP_2_ antagonist (CAY10471) were added up into the intervention group at the concentration of 10^−5^ M, respectively for 24 hours at 37 °C and 5% CO_2_. The culture medium was removed, Fluo-3/AM was labeled with a fluorescent Ca^2+^ probe, the intensity of Ca^2+^ fluorescence was observed and measured with a laser scanning confocal microscope (*Bio-Rad, USA*). At the same time, 10 cells were randomly selected in the uniformity field of the fluorescence intensity to analyze the fluorescence intensity.

### Statistical analysis

Data were presented as mean ± SD. All data were analyzed with SPSS 12.0 (*SPSS Inc. Chicago, US*) unless otherwise indicated. For the content of PGD_2_, statistical significance was determined by Student’s *t* test for pairwise comparisons. For RT-PCR, WB, LDH and MTT data, statistical significance was determined by one-way ANOVA with Dunnett’s multiple comparisons. *p* < 0.05 was considered statistically significant.

## Additional Information

**How to cite this article**: Ma, J. *et al*. Effect of the PGD_2_-DP signaling pathway on primary cultured rat hippocampal neuron injury caused by aluminum overload. *Sci. Rep.* **6**, 24646; doi: 10.1038/srep24646 (2016).
